# A prediction model and risk stratification tool for survival by chemotherapy in invasive micropapillary carcinoma of the breast: a population-based study with external validation

**DOI:** 10.3389/fonc.2026.1746971

**Published:** 2026-06-04

**Authors:** Fang Yang, Yiqi Yang, Qiuping Xu, Lin Tang, Yucai Wang, Elizabeth J. Cathcart-Rake, Li Xie

**Affiliations:** 1 The Comprehensive Cancer Center of Nanjing Drum Tower Hospital, Affiliated Hospital of Medical School, Nanjing University, Nanjing, China; 2 Department of Rheumatology and Immunology, Jinling Hospital, Affiliated Hospital of Medical School, Nanjing University, Nanjing, China; 3 Division of Hematology, Mayo Clinic, Rochester, MN, United States; 4 Department of Oncology, Mayo Clinic, Rochester, MN, United States

**Keywords:** chemotherapy, invasive micropapillary carcinoma, overall survival, prognosis, risk score

## Abstract

**Background:**

Invasive micropapillary carcinoma (IMPC) of the breast is associated with a high propensity for lymphovascular invasion and lymph node metastasis. However, whether chemotherapy is associated with improved survival outcomes in these patients remains unclear.

**Methods:**

This retrospective study analyzed IMPC patients from the SEER database (2010–2018) (n=1,150) and an external validation cohort from Nanjing Drum Tower Hospital (n=199). Propensity score matching assessed chemotherapy’s impact on overall survival (OS) and breast cancer-specific survival (BCSS) in the SEER cohort. A risk stratification model was developed and validated using the external cohort.

**Results:**

In the SEER cohort, chemotherapy was associated with significantly improved OS (HR = 0.517, 95% CI 0.293-0.910, *P* = 0.020) but not BCSS. A risk model incorporating marital status, tumor size, subtype, and radiotherapy history identified high- and low-risk groups. Chemotherapy was associated with significantly better OS only for high-risk patients (internal training cohort: HR = 0.577, 95% CI 0.349-0.952, *P* = 0.029; internal validation cohort: HR = 0.213, 95% CI 0.070-0.647, *P* = 0.003). External validation consistently confirmed poorer prognosis for high-risk patients (HR = 2.969, 95% CI 1.028-8.571, *P* = 0.035) and verified the model’s ability to identify patients with differential OS associations with chemotherapy: chemotherapy was associated with better OS in high-risk patients (HR = 0.163, 95% CI 0.031-0.868, *P* = 0.016), but not in low-risk patients (HR = 0.793, 95% CI 0.153-4.103, *P* = 0.782).

**Conclusions:**

Chemotherapy was associated with improved prognosis particularly in high-risk patients with IMPC. However, chemotherapy decisions should be made cautiously for low-risk patients, taking into account a comprehensive assessment of clinical and pathological factors.

## 1 Introduction

Invasive micropapillary carcinoma (IMPC), first described by Fisher et al. in 1980 (1) and subsequently characterized by Siriaunkgul and Tavassoli in 1993 (2), was formally classified as a distinct subtype in the 2003 World Health Organization (WHO) classification of breast tumors (3). IMPC is a relatively rare histologic subtype, accounting for approximately 1.0% to 8.4% of all invasive breast cancers (4–7). Despite its rarity, IMPC exhibits aggressive biological behavior with frequent lymphovascular invasion (LVI) and lymph node metastasis (LNM) (8–10), often presenting at advanced stages, which are associated with worse survival outcomes (11). Pathologically, IMPC demonstrates unique hollow cell clusters arranged in an “inside-out” pseudopapillary pattern (12). This distinct profile complicates therapeutic decisions. Currently, no IMPC−specific treatment guidelines exist. Consequently, clinical management of IMPC largely follows principles adapted from those for invasive ductal carcinoma (IDC), the most common type of breast cancer.

Chemotherapy is standard for high−risk breast cancer, which is conventionally defined by large tumor size, positive lymph nodes, high histological grade, or aggressive molecular subtypes such as triple−negative or human epidermal growth factor receptor 2-positive. However, whether chemotherapy is associated with improved survival specifically for the IMPC subtype remains uncertain (7). Recent evidence on neoadjuvant chemotherapy suggests that IMPC exhibits limited tumor response, with low pathologic complete response rates compared with invasive breast carcinoma of no special type (13), particularly in HER2−positive patients (14). In contrast, data on adjuvant chemotherapy in IMPC remain sparse. To date, no specialized predictive model for chemotherapy−associated survival exists specifically for IMPC patients. Given the substantial biological differences between IMPC and IDC, particularly the higher propensity for LVI and nodal metastasis in IMPC, applying IDC−derived treatment algorithms may not be optimal for IMPC patients. The absence of IMPC−specific predictive tools may result in either overtreatment or undertreatment.

Therefore, the aim of this study is to identify patients who are most likely to benefit from chemotherapy. Using data from the Surveillance, Epidemiology, and End Results (SEER) database, we developed a risk-stratified prediction model with a nomogram, based on the clinicopathological features of patients with IMPC. To ensure the reliability and clinical applicability of our model, we performed external validation with an independent patient cohort. This model is designed to inform and optimize individualized treatment decisions, ultimately improving patient outcomes.

## 2 Methods

### 2.1 Study design and patient selection

This retrospective study utilized data from two independent cohorts. The primary data source was the SEER database from 2010 to 2018, extracted using SEER*Stat software (version 8.4.3). The external validation cohort consisted of patients diagnosed between 2010 and 2025 from Nanjing Drum Tower Hospital. Patients who met the following inclusion criteria were selected for both cohorts (1): female (2); age ≥18 years at diagnosis (3); no distant metastasis at diagnosis (M0) (4); histologically confirmed invasive breast carcinoma (5); primary site confined to the breast; and (6) coded as invasive micropapillary carcinoma (ICD-O-3 code 8507).

Exclusion criteria for both cohorts included patients with (1): missing data on race, marital status, pathological grade, tumor size, lymph node status, estrogen receptor (ER) status, progesterone receptor (PR) status, or HER2 status (2); multiple primary tumors (including bilateral breast cancer) (3); diagnosis based on autopsy or death certificate only; and (4) inadequate follow-up information, defined as follow-up time of zero months or missing survival data. After applying these criteria, a cohort of eligible patients was selected for analysis.

The SEER database is a publicly available, de−identified cancer registry, and informed consent was not required per its data use agreement. The external validation cohort was approved by the Medical Ethics Committee of Nanjing Drum Tower Hospital. Due to the retrospective nature and de−identified data, informed consent was waived.

### 2.2 Statistical analysis

#### 2.2.1 Propensity score matching and covariate selection

To balance baseline characteristics between the chemotherapy and non-chemotherapy groups and mitigate confounding bias, PSM was employed in the SEER cohort. A 1:1 nearest-neighbor matching algorithm was used, with a caliper set at 0.2 times the standard deviation of the logit of the propensity score. The following covariates were included in the PSM model: age (< 50 years, ≥ 50 years), race (White, Black, Others), marital status (married, not married), laterality (left, right), histological grade (I−II, III−IV), tumor size (T1, T2, T3, T4), lymph node status (N0, N1, N2, N3), ER status (negative, positive), PR status (negative, positive), HER2 status (negative, positive), molecular subtype (HR+HER2−, HR+HER2+, HR−HER2+, HR−HER2−), surgery history (no, yes), and radiotherapy history (no, yes). The 50−year cutoff for age was selected because it has been used in previous SEER−based IMPC studies (15) and approximates the median age at diagnosis of IMPC (11). The balance of baseline characteristics between the two groups after matching was assessed using Chi-square tests.

#### 2.2.2 Definition of chemotherapy exposure and survival analyses

Chemotherapy exposure was defined based on the SEER−recorded binary variable, which indicates whether a patient received chemotherapy at any time after diagnosis. The exact start date of chemotherapy is not available in SEER. Univariate and multivariate Cox regression analyses were conducted in the PSM−matched cohort to identify independent prognostic factors associated with overall survival (OS) and breast cancer−specific survival (BCSS). For BCSS, death due to breast cancer was defined as the event and deaths from other causes were treated as censored observations. The multivariable Cox models included the following covariates: age, race, marital status, laterality, histological grade, tumor size, lymph node status, molecular subtype, surgery, and radiotherapy. In the univariate analysis, variables with a *P* value < 0.05 were included in the multivariate analysis to determine independent predictors of OS.

#### 2.2.3 Nomogram development and validation

The selected independent prognostic factors are established determinants with prognostic value independent of whether patients received chemotherapy. To preserve adequate sample size for model development, we applied them to the full SEER cohort rather than the smaller PSM−matched cohort. For the construction and validation of the prediction model, the SEER dataset was randomly split into training and validation sets in a 7:3 ratio using simple random sampling implemented in R. Using the training set, a nomogram was developed based on the selected independent prognostic factors. Points in the nomogram were derived by linear transformation of the regression coefficients from the multivariable Cox model, preserving the relative prognostic weight of each factor. Model performance was evaluated using multiple methods, including receiver operating characteristic (ROC) curve analysis, calibration curves, and decision curve analysis. The risk score formula was derived from the nomogram.

#### 2.2.4 Risk stratification and X-tile optimization

For dynamic risk stratification, X−tile software (version 3.6.1) selected the optimal cut−off by maximizing the log−rank test statistic across all possible threshold values of the risk score (16). The optimal cut−off was determined on the SEER cohort and then applied to the external validation cohort to test its generalizability. Patients were categorized into model−defined high-risk (risk score > cutoff) and low-risk (risk score ≤ cutoff) groups. Kaplan-Meier survival analysis was then performed to assess survival differences between these risk groups and evaluate the benefit of chemotherapy in each subgroup.

#### 2.2.5 Statistical software and significance

All *P* values were two-sided, and a *P* value < 0.05 was considered statistically significant. These analyses were performed using SPSS software (version 21) and R software (version 4.4.1).

## 3 Results

### 3.1 Patient characteristics

A total of 1,150 patients with IMPC from the SEER database were included in the primary analysis, with a median follow-up duration of 54.0 months. An external validation cohort comprising 199 patients from Nanjing Drum Tower Hospital was also analyzed, with a median follow-up of 23.0 months. The sample sizes of both cohorts align with the reported incidence range of IMPC (4–7), supporting the representativeness of our study population. The demographic and clinicopathological characteristics of the SEER cohort are summarized in **Table 1**, while those of the external cohort are presented in **Supplementary Table 1**.

**Table 1:** Characteristics of patients with IMPC from SEER database before and after PSM.

Variables		Before PSM				After PSM		
	Case, n	No chemotherapy, n (%)	Chemotherapy, n (%)	*P* value	Case, n	No chemotherapy, n (%)	Chemotherapy, n (%)	*P* value
No. of patients	1150	543	607		474	237	237	
Age (years)				0.000				0.553
<50	247	53 (9.761)	194 (31.960)		87	41 (17.300)	46 (19.409)	
>=50	903	490 (90.239)	413 (68.040)		387	196 (82.700)	191 (80.591)	
Race				0.170				0.539
White	885	424 (78.085)	461 (75.947)		370	190 (80.169)	180 (75.949)	
Black	122	48 (8.840)	74 (12.191)		49	22 (9.283)	27 (11.392)	
Others^‡^	143	71 (13.075)	72 (11.682)		55	25 (10.549)	30 (12.658)	
Marital status				0.048				0.516
Married	687	308 (56.722)	379 (62.438)		271	139 (58.650)	132 (55.696)	
Not married^φ^	463	235 (43.278)	228 (37.562)		203	98 (41.350)	105 (44.304)	
Laterality				0.113				0.519
Left	554	275 (50.645)	279 (45.964)		219	113 (47.679)	106 (44.726)	
Right	596	268 (49.355)	328 (54.036)		255	124 (52.321)	131 (55.274)	
Grade				0.000				0.444
I-II	753	425 (78.269)	328 (54.036)		304	156 (65.823)	148 (62.447)	
III-IV	397	118 (21.731)	279 (45.964)		170	81 (34.177)	89 (37.553)	
Tumor stage				0.000				0.180
T1	655	394 (72.560)	261 (42.998)		246	130 (54.852)	116 (48.945)	
T2	372	128 (23.573)	244 (40.198)		182	90 (37.975)	92 (38.819)	
T3	93	17 (3.131)	76 (12.521)		31	13 (5.485)	18 (7.595)	
T4	30	4 (0.736)	26 (4.283)		15	4 (1.688)	11 (4.641)	
Nodal status				0.000				0.320
N0	589	413 (76.059)	176 (28.995)		206	107 (45.148)	99 (41.772)	
N1	362	99 (18.232)	263 (43.328)		191	99 (41.772)	92 (38.819)	
N2	122	18 (3.315)	104 (17.133)		44	18 (7.595)	26 (10.970)	
N3	77	13 (2.394)	64 (10.544)		33	13 (5.485)	20 (8.439)	
ER				0.000				0.253
Negative	90	17 (3.131)	73 (12.026)		41	17 (7.173)	24 (10.127)	
Positive	1060	526 (96.869)	534 (87.974)		433	220 (92.827)	213 (89.873)	
PR				0.000				0.130
Negative	188	50 (9.208)	138 (22.735)		91	39 (16.456)	52 (21.941)	
Positive	962	493 (90.792)	469 (77.265)		383	198 (83.544)	185 (78.059)	
HER2				0.000				0.402
Negative	884	504 (92.818)	380 (62.603)		389	198 (83.544)	191 (80.591)	
Positive	266	39 (7.182)	227 (37.397)		85	39 (16.456)	46 (19.409)	
Subtype				0.000				0.519
HR+HER2-	844	494 (90.976)	350 (57.661)		365	188 (79.325)	177 (74.684)	
HR+HER2+	220	34 (6.261)	186 (30.642)		71	34 (14.346)	37 (15.612)	
HR-HER2-	40	10 (1.842)	30 (4.942)		24	10 (4.219)	14 (5.907)	
HR-HER2+	46	5 (0.921)	41 (6.755)		14	5 (2.110)	9 (3.797)	
Radiotherapy				0.025				0.269
No	427	220 (40.516)	207 (34.102)		220	116 (48.945)	104 (43.882)	
Yes	723	323 (59.484)	400 (65.898)		254	121 (51.055)	133 (56.118)	
Surgery				0.392				0.815
No	33	18 (3.315)	15 (2.471)		19	9 (3.797)	10 (4.219)	
Yes	1117	525 (96.685)	592 (97.529)		455	228 (96.203)	227 (95.781)	

IMPC, invasive micropapillary carcinoma; SEER, Surveillance, Epidemiology, and End Results; PSM, propensity score matching; ER, estrogen receptor; PR, progesterone receptor; HER2, human epidermal growth factor receptor 2; HR, hormone receptor.

‡ Others included Asian or Pacific Islander, American Indian/Alaska Native, and unknown.

φ Not married includes divorced, separated, single (never married), unmarried, or domestic partner and widowed.

The analysis revealed that patients who received chemotherapy were significantly younger, had a higher proportion of married individuals, and exhibited higher histological grades, as well as more advanced tumor size and lymph node stages, compared to those who did not undergo chemotherapy. Furthermore, the chemotherapy group had a greater proportion of hormone receptor-negative and HER2-positve tumors, and a larger proportion of these patients received radiotherapy. However, no significant differences were observed between the two groups with regard to race, tumor laterality, or surgical treatment (**Table 1**).

**Figure 1:**
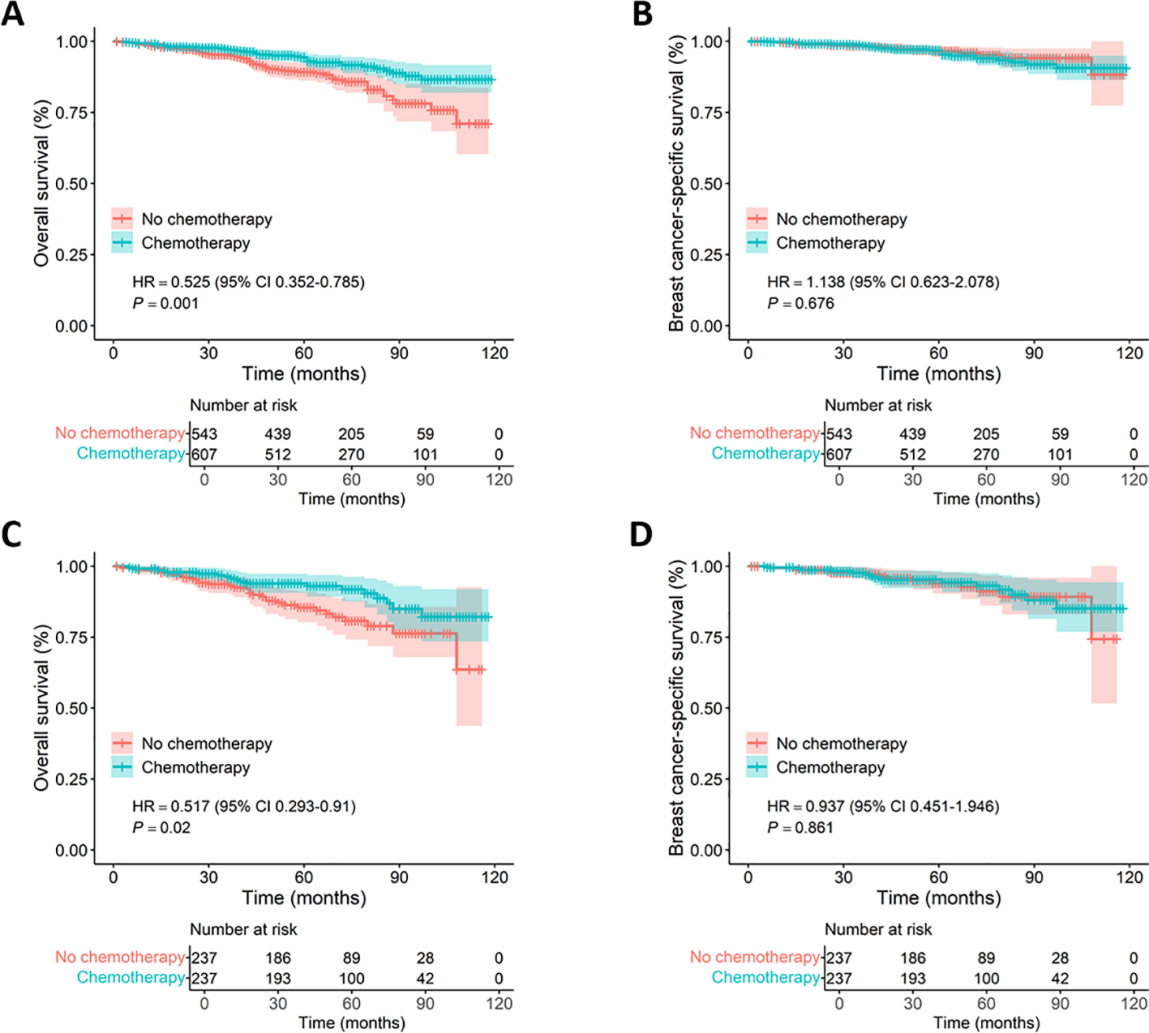
Kaplan-Meier curves of OS and BCSS of the chemotherapy and non-chemotherapy groups in the total SEER population before PSM **(A, B)** and after PSM **(C, D)**. OS: overall survival; BCSS: breast cancer-specific survival; PSM: propensity score matching.

Kaplan-Meier analysis of the SEER cohort showed that chemotherapy was associated with significantly improved OS compared to patients who did not receive chemotherapy (HR = 0.525, 95% CI 0.352 to 0.785, *P* = 0.001). However, no significant difference was observed in BCSS between the two groups (HR = 1.138, 95% CI 0.623 to 2.078, *P* = 0.676) (**Figures 1A, B**). After PSM to control for baseline differences, 474 matched patients were included, and the two groups showed no significant differences in baseline characteristics (**Table 1**). We analyzed the matched cohort and found that chemotherapy was associated with significantly better OS (HR = 0.517, 95% CI 0.293 to 0.910, *P* = 0.020), whereas no significant association was observed with BCSS (HR = 0.937, 95% CI 0.451 to 1.946, *P* = 0.861) (**Figures 1C, D**).

### 3.2 Independent prognostic factors

Univariate and multivariate Cox regression analyses of the PSM-matched SEER cohort identified several independent prognostic factors for OS. Marital status (HR = 2.529, 95% CI 1.404 to 4.557, *P* = 0.002), tumor size (T1 as reference, T4: HR = 6.630, 95% CI 2.399 to 18.323, *P* < 0.001), molecular subtype (HR+/HER2- as reference, HR-/HER2-: HR = 4.915, 95% CI 2.297 to 10.516, *P* < 0.001), and radiotherapy (HR = 0.558, 95% CI 0.314 to 0.991, *P* = 0.046) were found to be independent prognostic factors for OS. Specifically, unmarried patients, those with larger tumor size, triple-negative subtype, and those who did not receive radiotherapy had a worse prognosis. In contrast, age, race, tumor laterality, histological grade, lymph node stage, and surgical treatment were not significantly associated with OS (**Table 2**).

**Table 2:** Univariable and multivariable Cox regression analyses for predictive factors of OS after PSM.

Variables	Univariable analysis		Multivariable analysis	
	Hazard ratio (95% CI)	*P* value	Hazard ratio (95% CI)	*P* value
Age (years)				
<50	1 (reference)			
>=50	2.021 (0.863-4.734)	**0.105**		
Race				
White	1 (reference)			
Black	1.242 (0.524-2.939)	**0.623**		
Others^‡^	1.483 (0.691-3.184)	**0.312**		
Marital status				
Married	1 (reference)		1 (reference)	
Not married^φ^	2.928 (1.640-5.228)	**0.000**	2.529 (1.404-4.557)	**0.002**
Laterality				
Left	1 (reference)			
Right	1.380 (0.789-2.414)	**0.259**		
Grade				
I-II	1 (reference)			
III-IV	1.713 (0.992-2.959)	**0.054**		
Tumor stage				
T1	1 (reference)		1 (reference)	
T2	2.118 (1.149-3.905)	**0.016**	1.773 (0.945-3.327)	**0.074**
T3	1.832 (0.616-5.448)	**0.276**	1.535 (0.510-4.625)	**0.446**
T4	6.446 (2.373-17.512)	**0.000**	6.630 (2.399-18.323)	**0.000**
Nodal status				
N0	1 (reference)			
N1	1.380 (0.751-2.535)	**0.300**		
N2	1.076 (0.366-3.164)	**0.894**		
N3	2.142 (0.854-5.370)	**0.104**		
Subtype				
HR+HER2-	1 (reference)		1 (reference)	
HR+HER2+	1.471 (0.677-3.193)	**0.329**	1.316 (0.597-2.902)	**0.495**
HR-HER2-	5.075 (2.420-10.644)	**0.000**	4.915 (2.297-10.516)	**0.000**
HR-HER2+	1.628 (0.390-6.801)	**0.504**	2.451 (0.571-10.514)	**0.228**
Radiotherapy				
No	1 (reference)		1 (reference)	
Yes	0.495 (0.284-0.862)	**0.013**	0.558 (0.314-0.991)	**0.046**
Surgery				
No	1 (reference)			
Yes	0.492 (0.177-1.368)	**0.174**		

IMPC, invasive micropapillary carcinoma; OS, overall survival; PSM, propensity score matching; CI, confidence interval; HR, hormone receptor; HER2, human epidermal growth factor receptor 2.

‡ Others included Asian or Pacific Islander, American Indian/Alaska Native, and unknown.

φ Not married includes divorced, separated, single (never married), unmarried, or domestic partner and widowed.

### 3.3 Nomogram development and validation

**Figure 2:**
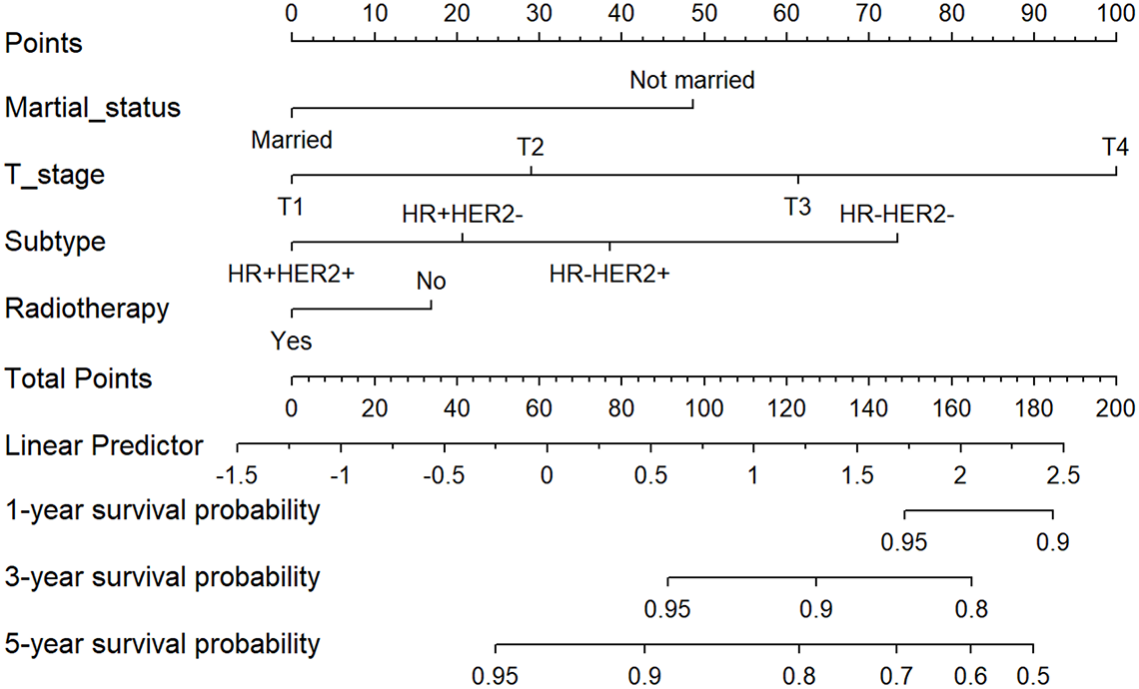
Nomogram for predicting 1-year, 3-year, and 5-year OS in patients with IMPC. OS, overall survival; IMPC, invasive micropapillary carcinoma.

**Figure 3:**
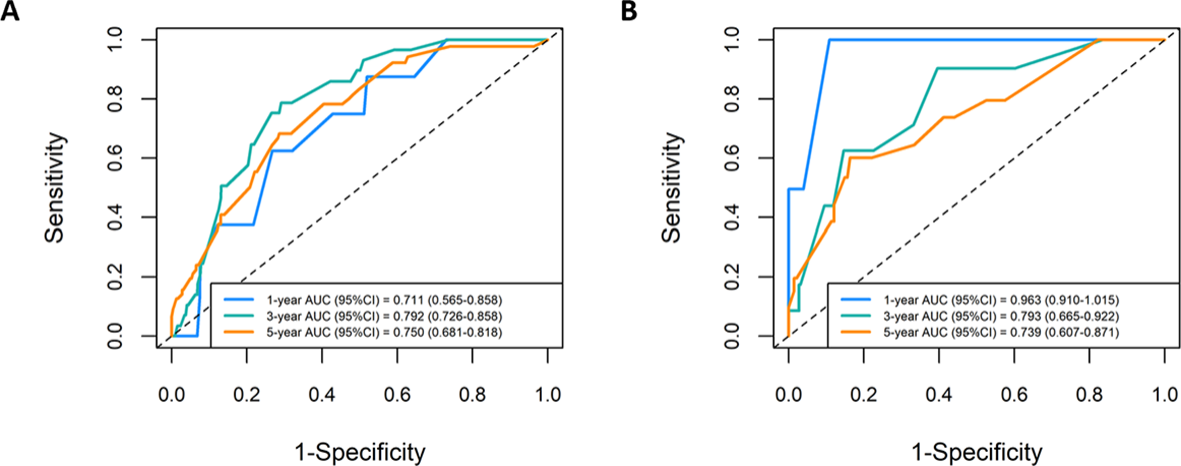
The ROC curves of 1-year, 3-year, and 5-year OS of the internal training cohort **(A)** and internal validation cohort **(B)**. ROC, receiver operating characteristic; AUC, area under the curve; OS, overall survival.

Patients from the SEER database were randomly divided into an internal training cohort and an internal validation cohort at a 7:3 ratio for model development (**Table 3**). In the training cohort, four independent prognostic factors for OS identified by Cox regression analysis were included in the nomogram to predict the 1-year, 3-year, and 5-year OS of patients with IMPC (**Figure 2**). Tumor size had the greatest impact on OS, followed by molecular subtype, marital status, and radiotherapy status. The discriminative ability of the nomogram was assessed using the area under the curve (AUC) of the ROC curve. In the training cohort, the AUC values for 1-year, 3-year, and 5-year OS were 0.711, 0.792, and 0.750, respectively (**Figure 3A**). In the validation cohort, the AUC values for 1-year, 3-year, and 5-year OS were 0.963, 0.793, and 0.739, respectively (**Figure 3B**). These results demonstrate that the nomogram exhibits high predictive accuracy. The calibration curves for both the training and validation cohorts showed strong concordance between the predicted and actual survival outcomes (**Supplementary Figure 1**). Additionally, decision curve analysis (DCA) revealed that the nomogram model provides significant net clinical benefit across a range of threshold probabilities (**Supplementary Figure 2**).

**Table 3:** Characteristics of patients with IMPC in training and validation cohorts.

Variables	Total (%)	Training (%)	Validation (%)	*P* value
No. of patients	1150	805	345	
Age (years)				0.541
<50	247 (21.478)	169 (20.994)	78 (22.609)	
>=50	903 (78.522)	636 (79.006)	267 (77.391)	
Race				0.774
White	885 (76.957)	622 (77.267)	263 (76.232)	
Black	122 (10.609)	82 (10.186)	40 (11.594)	
Others^‡^	143 (12.435)	101 (12.547)	42 (12.174)	
Marital status				0.352
Married	687 (59.739)	488 (60.621)	199 (57.681)	
Not married^φ^	463 (40.261)	317 (39.379)	146 (42.319)	
Laterality				0.979
Left	554 (48.174)	388 (48.199)	166 (48.116)	
Right	596 (51.826)	417 (51.801)	179 (51.884)	
Grade				0.989
I-II	753 (65.478)	527 (65.466)	226 (65.507)	
III-IV	397 (34.522)	278 (34.534)	119 (34.493)	
Tumor stage				0.370
T1	655 (56.957)	459 (57.019)	196 (56.812)	
T2	372 (32.348)	253 (31.429)	119 (34.493)	
T3	93 (8.087)	72 (8.944)	21 (6.087)	
T4	30 (2.609)	21 (2.609)	9 (2.609)	
Nodal status				0.520
N0	589 (51.217)	404 (50.186)	185 (53.623)	
N1	362 (31.478)	264 (32.795)	98 (28.406)	
N2	122 (10.609)	83 (10.311)	39 (11.304)	
N3	77 (6.696)	54 (6.708)	23 (6.667)	
ER				0.811
Negative	90 (7.826)	64 (7.950)	26 (7.536)	
Positive	1060 (92.174)	741 (92.050)	319 (92.464)	
PR				0.554
Negative	188 (16.348)	135 (16.770)	53 (15.362)	
Positive	962 (83.652)	670 (83.230)	292 (84.638)	
HER2				0.376
Negative	884 (76.870)	613 (76.149)	271 (78.551)	
Positive	266 (23.130)	192 (23.851)	74 (21.449)	
Subtype				0.804
HR+HER2-	844 (73.391)	584 (72.547)	260 (75.362)	
HR+HER2+	220 (19.131)	159 (19.752)	61 (17.681)	
HR-HER2-	40 (3.478)	29 (3.602)	11 (3.189)	
HR-HER2+	46 (4.000)	33 (4.099)	13 (3.768)	
Radiotherapy				0.497
No	427 (37.130)	304 (37.764)	123 (35.652)	
Yes	723 (62.870)	501 (62.236)	222 (64.348)	
Surgery				0.264
No	33 (2.870)	26 (3.230)	7 (2.029)	
Yes	1117 (97.130)	779 (96.770)	338 (97.971)	

IMPC, invasive micropapillary carcinoma; OS, overall survival; PSM, propensity score matching; ER, estrogen receptor; PR, progesterone receptor; HER2, human epidermal growth factor receptor 2.

‡ Others included Asian or Pacific Islander, American Indian/Alaska Native, and unknown.

φ Not married includes divorced, separated, single (never married), unmarried, or domestic partner and widowed.

### 3.4 Risk stratification analysis

**Figure 4:**
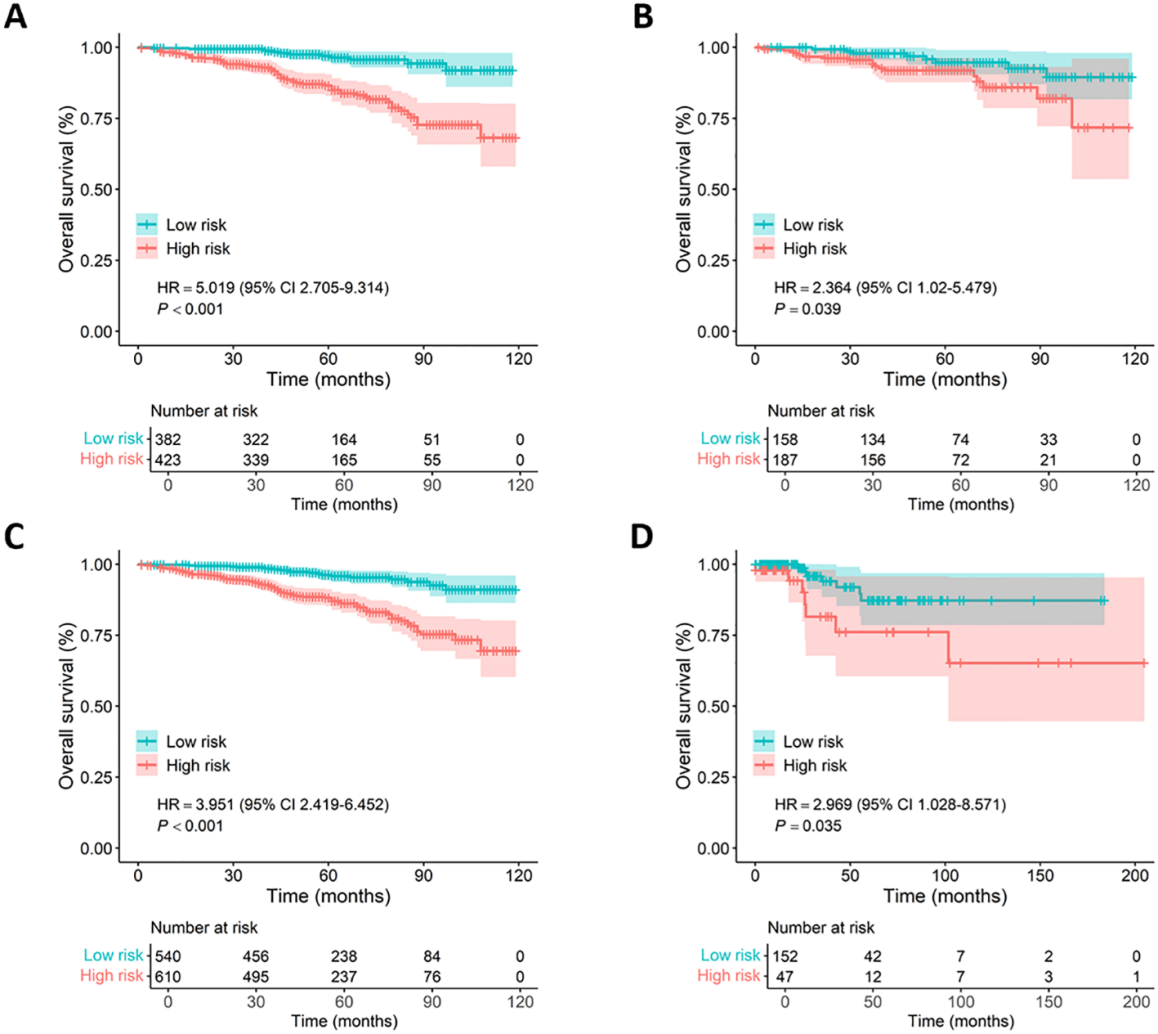
Kaplan-Meier curves of OS for IMPC patients stratified into low- and high-risk groups in the internal training cohort **(A)**, internal validation cohort **(B)**, total SEER population **(C)**, and external validation cohort **(D)**. OS, overall survival; IMPC, invasive micropapillary carcinoma.

We assigned scores to each of the four independent prognostic factors based on the nomogram (**Table 4**), and the total score was calculated for each patient in the internal training cohort to determine their risk score. The X-tile software was used to identify the optimal cutoff value for the risk score. Patients were classified into model−defined low-risk (risk score ≤ 50) and high-risk (risk score > 50) groups. Kaplan-Meier survival analysis demonstrated significantly better OS for low-risk patients compared to high-risk patients in the internal training cohort (HR = 5.019, 95% CI 2.705 to 9.314, *P* < 0.001), internal validation cohort (HR = 2.364, 95% CI 1.020 to 5.479, *P* = 0.039), and the entire SEER cohort (HR = 3.951, 95% CI 2.419 to 6.452, *P* < 0.001) (**Figures 4A–C**). Furthermore, this risk stratification was successfully validated in the external cohort, where high-risk patients showed significantly poorer prognosis compared to low-risk patients (HR = 2.969, 95% CI 1.028 to 8.571, *P* = 0.035) (**Figure 4D**). These consistent results across all cohorts indicate that the risk scoring system accurately predicts patient OS. The cut−off value derived from the SEER cohort was validated in the external cohort, supporting its generalizability.

**Table 4:** The risk score of each independent prognostic factor.

Characteristics	Points
Marital status	
Married	0
Not married^φ^	49
Tumor stage	
T1	0
T2	29
T3	61
T4	100
Subtype	
HR+HER2-	21
HR+HER2+	0
HR-HER2-	73
HR-HER2+	39
Radiotherapy	
No	17
Yes	0

HR, hormone receptor; HER2, human epidermal growth factor receptor 2.

φ Not married includes divorced, separated, single (never married), unmarried, or domestic partner and widowed.

### 3.5 Chemotherapy’s impact on survival by risk stratification

**Figure 5:**
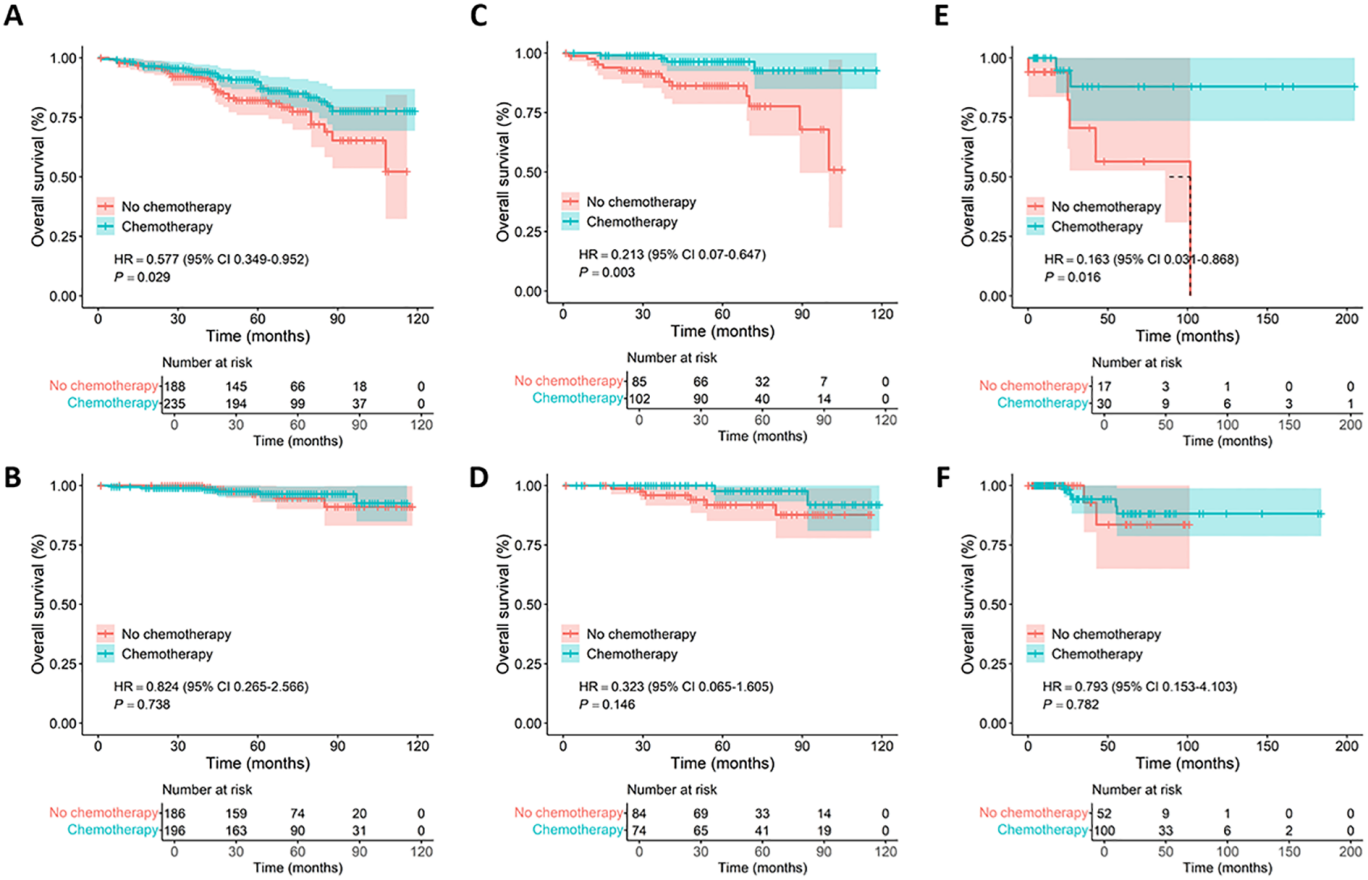
Kaplan-Meier curves of OS for IMPC patients stratified by high-risk and low-risk groups in internal training cohort **(A, B)**, internal validation cohort **(C, D)**, and external validation cohort **(E, F)**. OS, overall survival; IMPC, invasive micropapillary carcinoma.

Stratified analysis by risk level revealed consistent differences in the association between chemotherapy and OS across all study cohorts. In the internal training cohort, model−defined high-risk patients treated with chemotherapy showed significantly better OS (HR = 0.577, 95% CI 0.349-0.952, *P* = 0.029), while no significant advantage was observed in low-risk patients (HR = 0.824, 95% CI 0.265-2.566, *P* = 0.738) (**Figures 5A, B**). Similarly, in the internal validation cohort, chemotherapy was associated with significantly better OS in high-risk patients (HR = 0.213, 95% CI 0.070-0.647, *P* = 0.003), whereas no significant association was observed in low-risk patients (HR = 0.323, 95% CI 0.065-1.605, *P* = 0.146) (**Figures 5C, D**). This differential association pattern was further confirmed in the external validation cohort, where chemotherapy was associated with significantly better OS in high-risk patients (HR = 0.163, 95% CI 0.031-0.868, *P* = 0.016), but no significant association was observed in low-risk patients (HR = 0.793, 95% CI 0.153-4.103, *P* = 0.782) (**Figures 5E, F**). The consistency of these findings across all cohorts strongly supports the clinical utility of our risk stratification model in identifying IMPC patients in whom chemotherapy is associated with better OS.

## 4 Discussion

IMPC exhibits distinct cellular morphology and biological behavior versus IDC, including larger tumors, advanced staging, and frequent nodal involvement despite higher HR-positivity (17). Genomically, it demonstrates monoclonal metastasis with copy-number-altered subclones (18, 19), KRT80-mediated metabolic heterogeneity (20), and PRMT1/3 epigenetic dysregulation (21, 22), and SREBF1-driven lipid metabolic reprogramming (23), collectively underpinning its aggressive phenotype. Despite its aggressive features, IMPC paradoxically shows better long-term survival than IDC (5, 24), potentially due to reduced pro-oncogene expression (25), although conflicting data exist (9, 11, 26, 27). Chemotherapy benefit in IMPC remains disputed (4, 28–30). To address this gap, we developed the first risk stratification model specifically for IMPC. Using PSM to control for confounding but without adjusting for matched-pair correlation, we identified four prognostic factors based on prior clinical knowledge. The training and validation split was used solely for performance assessment, reducing the risk of overfitting. Our findings indicate that chemotherapy is associated with significantly better OS in high-risk patients, while no such association is observed in low-risk patients. This personalized risk scoring system can guide clinical decision-making by identifying high−risk patients likely to have improved OS associated with chemotherapy and sparing low-risk patients from unnecessary treatment, thereby minimizing treatment-related burdens and side effects.

A noteworthy finding in our study is the discordance between OS and BCSS. In the SEER cohort, chemotherapy was significantly associated with improved OS but not with BCSS. Several factors may explain this inconsistency. First, chemotherapy−related non−cancer toxicities, including cardiotoxicity, infections, and second malignancies, affect OS but are excluded from BCSS, potentially masking a modest cancer−specific benefit. Second, the much smaller number of BCSS events reduces statistical power to detect a modest effect. Third, the observed OS benefit could partly reflect unmeasured confounding, such as more frequent follow−up or better management of comorbidities in the chemotherapy group, rather than a direct anti−cancer effect. We attempted a Fine−Gray competing−risks model for BCSS, but stable estimates could not be obtained due to the limited number of breast cancer−specific deaths (n = 44) relative to the number of covariates. Under these conditions, the competing−risks model may fail to converge or yield unreliable results. Thus, we report conventional Cox regression for BCSS, given that the low event rate minimizes bias from competing risks. In the SEER cohort, chemotherapy was not significantly associated with BCSS (**Supplementary Table 2**).

We further performed a formal treatment−by−risk interaction test to evaluate whether the association between chemotherapy and OS differed by risk group. In the total SEER cohort, after adjusting for age, race, marital status, tumor size, lymph node status, molecular subtype, and radiotherapy history, the interaction term was not statistically significant (*P* = 0.712). In contrast, in the independent external validation cohort, after adjusting for the same covariates except race, the interaction term reached statistical significance (*P* = 0.048). The discrepancy between the two cohorts may be partly attributable to the fact that the external validation cohort consisted exclusively of Chinese patients, and race was therefore not included in the model, whereas the SEER cohort included diverse racial groups. Given the inconsistent interaction results, our subgroup findings should be interpreted as exploratory, and further validation in large−scale, prospective cohorts with longer follow−up is warranted.

Our findings indicate a limited association between chemotherapy and better OS in low-risk patients with IMPC, which may be explained by several factors. Firstly, the indolent biological behavior of low-risk IMPC inherently confers excellent survival outcomes, reducing the marginal utility of cytotoxic intervention. Secondly, the diminished proliferative capacity characteristic of low-risk tumors potentially undermines chemotherapy effectiveness, which relies on targeting mitotically active cells. Thirdly, the predominant HR-positive status observed in our IMPC cohort indicates that most patients likely underwent endocrine therapy. The established clinical efficacy of endocrine regimens may consequently reduce the survival-enhancing potential of chemotherapy through effective estrogen signaling suppression.

Recent evidence suggests that IMPC may be relatively resistant to neoadjuvant therapy, particularly anti−HER2 regimens (13, 14). In our study, we did not distinguish between neoadjuvant and adjuvant chemotherapy, as our primary objective was to evaluate the overall association between chemotherapy and survival and to develop a risk stratification model for IMPC, rather than to compare neoadjuvant versus adjuvant regimens. Similarly, we did not differentiate between breast−conserving surgery and mastectomy. Future studies with detailed treatment information are warranted to clarify these issues. Our analysis did not incorporate information on anti−HER2 therapy, even though in clinical practice HER2−positive breast cancer patients receiving chemotherapy almost always receive concurrent HER2−directed agents with proven survival benefits. Therefore, the observed OS benefit in HER2−positive subgroups could indeed be partly attributable to targeted therapy rather than chemotherapy alone. To explore this possibility, we performed a post−hoc analysis restricted to HER2−negative patients, who would not have received anti−HER2 agents. The results were consistent with the main findings. In the overall HER2−negative population, chemotherapy was associated with improved OS (HR = 0.601, 95% CI 0.379−0.954, *P* = 0.029). Risk stratification further showed that high−risk HER2−negative patients showed a significant association between chemotherapy and improved OS (HR = 0.573, 95% CI 0.345−0.950, *P* = 0.029), whereas low−risk HER2−negative patients did not (HR = 0.450, 95% CI 0.142−1.427, *P* = 0.164) (**Supplementary Figure 3**). These findings suggest that the observed association between chemotherapy and OS in our risk stratification model may not be solely attributable to unmeasured anti−HER2 therapy.

Consistent with prior studies, marital status, tumor size, molecular subtype, and radiotherapy were identified as independent prognostic determinants (4, 28–31). The external validation cohort demonstrated a higher proportion of low-risk patients compared to the SEER cohorts, which likely reflects its notably higher percentage of married individuals-a favorable prognostic factor in our risk model. Marital status may influence OS through several mechanisms. Married patients often benefit from greater financial resource and enhanced treatment adherence support. Unmarried status may correlate with reduced access to timely medical interventions. Crucially, this association reflects non-causal correlation rather than biological determinism. Comprehensive social support parameters, rather than marital status alone, should be integrated into clinical frameworks to mitigate ethical concerns and prevent care disparities. Other reported prognostic determinants vary across studies, including age (4, 29, 30, 32, 33), race (4, 28), histological grade (31), nodal involvement (28, 32, 34, 35), and surgery (28–30). These discrepancies may be due to differences in grouping criteria, statistical methods, and database updates.

Our study has several important strengths. First, we developed the first risk-stratification framework specifically designed to guide chemotherapy decisions in IMPC, integrating key clinicopathological and molecular features to objectively classify patients into distinct prognostic groups. Second, by utilizing the extensive SEER registry we assembled a large IMPC cohort and further validated the model through an external cohort from a different healthcare setting, thereby addressing statistical challenges of this rare malignancy while confirming the model’s generalizability. Third, we employed PSM to systematically balance baseline characteristics between treatment groups, minimizing selection bias and enhancing the reliability of our findings.

Our study has several limitations. First, the retrospective cohort design introduces inherent selection bias, which may compromise the generalizability of our risk stratification model. Second, we did not include key clinical variables, including LVI status, performance status, details of endocrine therapy and HER2-directed therapy, chemotherapy regimen dose intensity, treatment completion, and comorbidities. These unmeasured confounders limit the precise estimation of treatment effects and may bias the observed association between chemotherapy and survival. Third, the study lacked detailed molecular characterization, which could provide further insights into the underlying mechanisms driving IMPC aggressiveness. Fourth, the external validation cohort had a shorter follow−up and a different time window than the SEER cohort, which may introduce residual confounding and limit the validation of long−term survival predictions. Additionally, the lack of chemotherapy start dates in SEER may introduce immortal time bias, potentially overestimating the associated survival benefit.

## 5 Conclusion

This study introduces a novel risk stratification model to assess survival associated with chemotherapy in patients with IMPC, based on four independent prognostic factors. The findings suggest that IMPC−specific high-risk patients should receive chemotherapy, while its use should be cautious for low-risk patients. However, these results require validation in prospective studies to confirm their applicability and accuracy. Further research into the molecular and genomic characteristics of IMPC, as well as the impact of other treatment modalities, is crucial for refining treatment strategies and improving patient outcomes.

## Data Availability

The original contributions presented in the study are included in the article/**Supplementary Material**. Further inquiries can be directed to the corresponding authors.

## Supplementary material

Supplementary Figure 1 The calibration curves of nomogram for predicting 1-year, 3-year and 5-year OS in the internal training cohort **(A–C)**, and in the internal validation cohort **(D–F)**. The x-axis indicates the nomogram-predicted survival probability, and the y-axis indicates the actual survival probability. The dashed line indicates that the prediction agrees with actuality. OS, overall survival.

Supplementary Figure 2 Decision curve analysis for the nomogram in predicting 1−, 3−, and 5−year overall survival in the training **(A–C)** and validation **(D–F)** cohorts. Blue: nomogram; yellow: treat−all; black: treat−none. The nomogram provided positive net benefit over threshold probabilities of 0-0.03 (1-year), 0-0.1 (3-year), and 0-0.6 (5-year) in the training cohort (maximum net benefit: 0.01, 0.04, and 0.07, respectively), and over 0-0.2 (1-year), 0-0.6 (3-year), and 0-0.8 (5-year) in the validation cohort (maximum net benefit: 0.01, 0.03, and 0.06, respectively).

Supplementary Figure 3 Kaplan-Meier curves of OS in HER2−negative IMPC patients from the SEER cohort for overall population **(A)**, high−risk subgroup **(B)**, and low−risk subgroup **(C)**. OS, overall survival; IMPC, invasive micropapillary carcinoma; HER2, human epidermal growth factor receptor 2.
