# Supplementary figures and images for: A prediction model and risk stratification tool for survival by chemotherapy in invasive micropapillary carcinoma of the breast: a population-based study with external validation
Source: Front Oncol. 2026 Jun 4;16:1746971. doi: 10.3389/fonc.2026.1746971 (PMC13275252; doi:10.3389/fonc.2026.1746971)

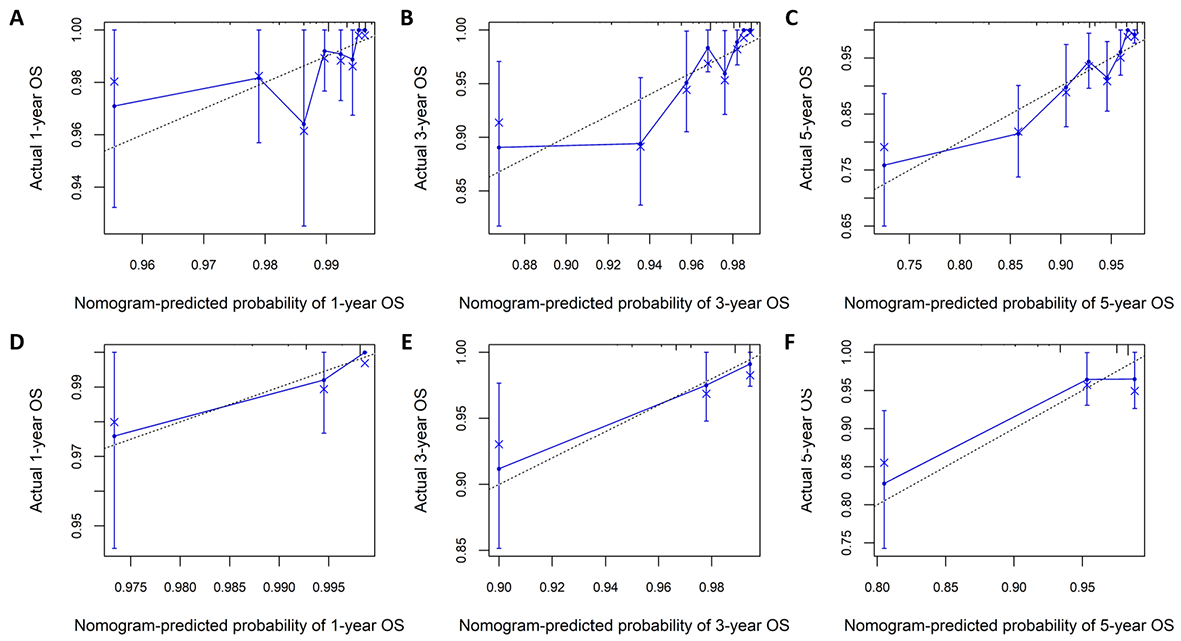

Supplement: Supplementary Figure 1 — The calibration curves of nomogram for predicting 1-year, 3-year and 5-year OS in the internal training cohort (A–C), and in the internal validation cohort (D–F). The x-axis indicates the nomogram-predicted survival probability, and the y-axis indicates the actual survival probability. The dashed line indicates that the prediction agrees with actuality. OS, overall survival. [file Image1.tif]

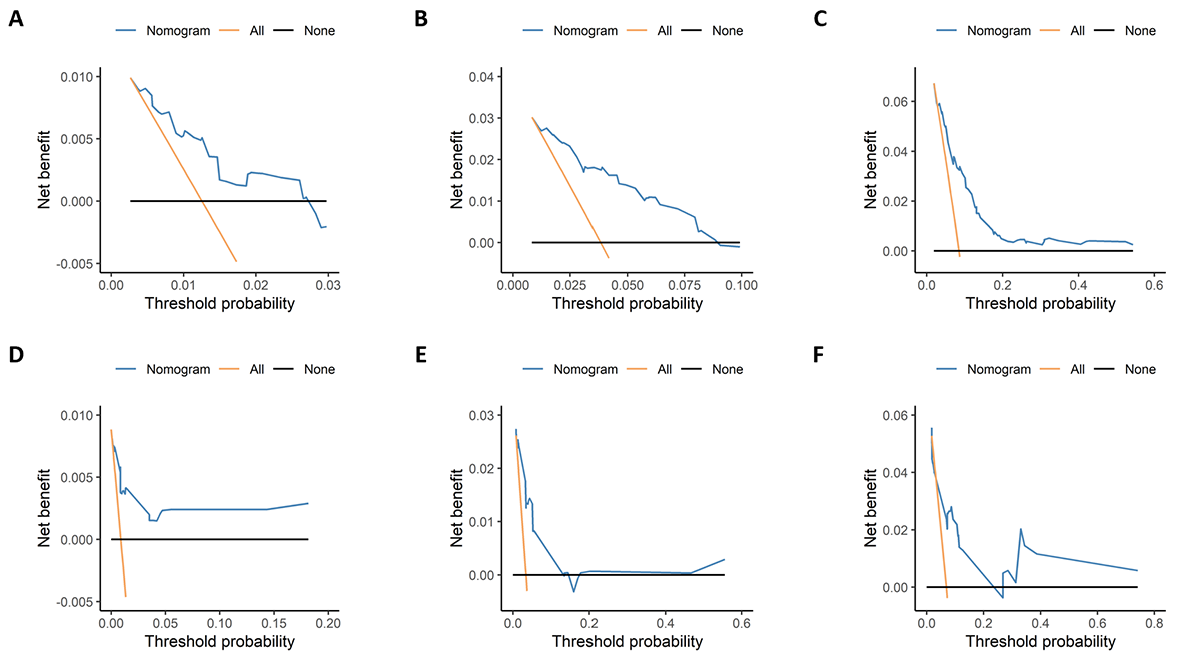

Supplement: Supplementary Figure 2 — Decision curve analysis for the nomogram in predicting 1−, 3−, and 5−year overall survival in the training (A–C) and validation (D–F) cohorts. Blue: nomogram; yellow: treat−all; black: treat−none. The nomogram provided positive net benefit over threshold probabilities of 0-0.03 (1-year), 0-0.1 (3-year), and 0-0.6 (5-year) in the training cohort (maximum net benefit: 0.01, 0.04, and 0.07, respectively), and over 0-0.2 (1-year), 0-0.6 (3-year), and 0-0.8 (5-year) in the validation cohort (maximum net benefit: 0.01, 0.03, and 0.06, respectively). [file Image2.tif]
