# Supplementary material for: A prediction model and risk stratification tool for survival by chemotherapy in invasive micropapillary carcinoma of the breast: a population-based study with external validation
Source: Front Oncol. 2026 Jun 4;16:1746971. doi: 10.3389/fonc.2026.1746971 (PMC13275252; doi:10.3389/fonc.2026.1746971)
Supplement: Supplementary file 4 [file Table1.docx]

**TABLE S1** Characteristics of patients with IMPC from external validation cohort (n=199).

| **Variables** | **n (%)** |
| --- | --- |
| No. of patients | 199 (100.000) |
| Age (years) |  |
| <50 | 55 (27.638) |
| >=50 | 144 (72.362) |
| Marital status |  |
| Married | 188 (94.472) |
| Not married^φ^ | 11 (5.528) |
| Laterality |  |
| Left | 106 (53.266) |
| Right | 93 (46.734) |
| Grade |  |
| I-II | 147 (73.869) |
| III-IV | 52 (26.131) |
| Tumor stage |  |
| T1 | 91 (45.729) |
| T2 | 97 (48.744) |
| T3 | 9 (4.522) |
| T4 | 2 (1.005) |
| Nodal status |  |
| N0 | 75 (37.689) |
| N1 | 66 (33.166) |
| N2 | 34 (17.085) |
| N3 | 24 (12.060) |
| Subtype |  |
| HR+HER2- | 141 (70.854) |
| HR+HER2+ | 43 (21.608) |
| HR-HER2- | 7 (3.518) |
| HR-HER2+ | 8 (4.020) |
| Chemotherapy |  |
| No | 69 (34.673) |
| Yes | 130 (65.327) |
| Radiotherapy |  |
| No | 71 (35.678) |
| Yes | 128 (64.322) |

^φ^ Not married includes divorced, separated, single (never married), unmarried, or domestic partner and widowed.
