# Supplementary material for: A prediction model and risk stratification tool for survival by chemotherapy in invasive micropapillary carcinoma of the breast: a population-based study with external validation
Source: Front Oncol. 2026 Jun 4;16:1746971. doi: 10.3389/fonc.2026.1746971 (PMC13275252; doi:10.3389/fonc.2026.1746971)
Supplement: Supplementary file 5 [file Table2.docx]

**TABLE S2** Univariable Cox regression analyses for predictive factors of BCSS before and after PSM.

| Variables | Before PSM | |  | After PSM | |
| --- | --- | --- | --- | --- | --- |
|  | Hazard ratio (95% CI) | *P* value |  | Hazard ratio (95% CI) | *P* value |
| Age (years) |  |  |  |  |  |
| <50 | 1 (reference) |  |  | 1 (reference) |  |
| >=50 | 1.006 (0.497-2.038) | 0.986 |  | 1.268 (0.483-3.326) | 0.630 |
| Race |  |  |  |  |  |
| White | 1 (reference) |  |  | 1 (reference) |  |
| Black | 1.410 (0.588-3.381) | 0.441 |  | 0.762 (0.179-3.255) | 0.714 |
| Others^‡^ | 1.432 (0.630-3.254) | 0.392 |  | 1.974 (0.794-4.908) | 0.143 |
| Marital status |  |  |  |  |  |
| Married | 1 (reference) |  |  | 1 (reference) |  |
| Not married^φ^ | 2.833 (1.532-5.238) | 0.001 |  | 2.706 (1.258-5.823) | 0.011 |
| Laterality |  |  |  |  |  |
| Left | 1 (reference) |  |  | 1 (reference) |  |
| Right | 1.515 (0.826-2.781) | 0.179 |  | 2.308 (1.021-5.219) | 0.044 |
| Grade |  |  |  |  |  |
| I-II | 1 (reference) |  |  | 1 (reference) |  |
| III-IV | 3.132 (1.706-5.749) | 0.000 |  | 2.487 (1.195-5.180) | 0.015 |
| Tumor stage |  |  |  |  |  |
| T1 | 1 (reference) |  |  | 1 (reference) |  |
| T2 | 2.807 (1.362-5.783) | 0.005 |  | 2.973 (1.212-7.296) | 0.017 |
| T3 | 6.124 (2.645-14.181) | 0.000 |  | 4.398 (1.286-15.045) | 0.018 |
| T4 | 7.934 (2.226-28.273) | 0.001 |  | 9.506 (2.451-36.872) | 0.001 |
| Nodal status |  |  |  |  |  |
| N0 | 1 (reference) |  |  | 1 (reference) |  |
| N1 | 1.444 (0.697-2.993) | 0.323 |  | 1.346 (0.602-3.010) | 0.469 |
| N2 | 1.552 (0.564-4.271) | 0.395 |  | 0.470 (0.061-3.644) | 0.470 |
| N3 | 4.797 (2.152-10.692) | 0.000 |  | 2.428 (0.771-7.644) | 0.130 |
| Subtype |  |  |  |  |  |
| HR+HER2- | 1 (reference) |  |  | 1 (reference) |  |
| HR+HER2+ | 0.931 (0.384-2.256) | 0.874 |  | 1.108 (0.323-3.797) | 0.870 |
| HR-HER2- | 7.016 (3.184-15.461) | 0.000 |  | 7.876 (3.243-19.123) | 0.000 |
| HR-HER2+ | 2.132 (0.646-7.040) | 0.214 |  | 3.097 (0.712-13.466) | 0.132 |
| Radiotherapy |  |  |  |  |  |
| No | 1 (reference) |  |  | 1 (reference) |  |
| Yes | 0.537 (0.297-0.970) | 0.039 |  | 0.582 (0.280-1.212) | 0.148 |
| Surgery |  |  |  |  |  |
| No | 1 (reference) |  |  | 1 (reference) |  |
| Yes | 0.164 (0.069-0.389) | 0.000 |  | 0.250 (0.087-0.724) | 0.011 |
| Chemotherapy |  |  |  |  |  |
| No | 1 (reference) |  |  | 1 (reference) |  |
| Yes | 1.138 (0.623-2.078) | 0.676 |  | 0.937 (0.451-1.946) | 0.861 |

Abbreviations: BCSS: breast cancer-specific survival; PSM: propensity score matching, RT: radiotherapy; CI confidence interval.

^‡^ Others included Asian or Pacific Islander, American Indian/Alaska Native. Unknown.

^φ^ Not married includes divorced, separated, single (never married), unmarried, or domestic partner and widowed.
